# Tumor-derived interleukin-1 receptor antagonist exhibits immunosuppressive functions and promotes pancreatic cancer

**DOI:** 10.1186/s13578-023-01090-8

**Published:** 2023-08-10

**Authors:** Yu-Ching Fan, Yu-Cin Fong, Chun-Tse Kuo, Chia-Wei Li, Wei-Yu Chen, Jian-Da Lin, Florian Bürtin, Michael Linnebacher, Quoc Thang Bui, Kuan-Der Lee, Yuan-Chin Tsai

**Affiliations:** 1 Ph.D. Program for Cancer Molecular Biology and Drug Discovery, College of Medical Science and Technology, Taipei Medical University and Academia Sinica, Taipei, Taiwan; 2 Graduate Institute of Cancer Biology and Drug Discovery, College of Medical Science and Technology, Taipei Medical University, Taipei, Taiwan; 3 Institute of Biomedical Sciences, Academia Sinica, Taipei, Taiwan; 4 Department of Pathology, Wan Fang Hospital, Taipei Medical University, Taipei, Taiwan; 5 Department of Pathology, School of Medicine, College of Medicine, Taipei Medical University, Taipei, Taiwan; 6 Department of Biochemical Science and Technology, College of Life Science, National Taiwan University, Taipei City, 10617 Taiwan; 7 Center for Computational and Systems Biology, National Taiwan University, Taipei City, 10617 Taiwan; 8 Clinic of General Surgery, University Medical Center Rostock, Schillingallee 35, 18057 Rostock, Germany; 9 Clinic of General Surgery, Molecular Oncology and Immunotherapy, University Medical Center Rostock, Schillingallee 69, 18057 Rostock, Germany; 10 International Ph.D. Program for Cell Therapy and Regeneration Medicine (IPCTRM), School of Medicine, College of Medicine, Taipei Medical University, Taipei, Taiwan; 11 Department of Post-Baccalaureate Medicine, College of Medicine, Natioanl Chung Hsing University, Taichung, Taiwan; 12 Cell Therapy and Regenerative Medicine Center and Comprehensive Cancer Center, Taichung Veterans General Hospital, Taichung, Taiwan; 13 Ph.D. Program for Cancer Molecular Biology and Drug Discovery, College of Medical Science and Technology, Taipei Medical University, Taipei, Taiwan

**Keywords:** Pancreatic adenocarcinoma, interleukin-1 receptor antagonist, Immunosuppression, Tumor microenvironment, IFN-γ, Patient-derived xenograft

## Abstract

**Background:**

Pancreatic ductal adenocarcinoma (PDA) is a pernicious disease characterized by an immunosuppressive milieu that is unresponsive to current immunotherapies. Interleukin-1 receptor antagonist (IL-1Ra) is a natural anti-inflammatory cytokine; however, its contribution to cancer pathogenesis and immunosuppression remains elusive. In this research, we investigated the role and mechanism of IL-1Ra in malignant progression of PDA.

**Results:**

Through analyzing clinical dataset and examining the pathological tumor tissues and serum samples, we have demonstrated that IL-1Ra expression is elevated in human PDA and positively associated with malignant progression of PDA. To study the biological function of IL-1Ra in tumors, we generated a set of mouse pancreatic cancer cell lines with a knockout (KO) of the *Il1rn* gene, encoding IL-1Ra, and compared the tumor growth rates in immune-competent and immune-deficient mice. We found that the *Il1rn* KO cells exhibited greater tumor inhibition in immune-competent mice, highlighting the crucial role of a functional immune system in *Il1rn* KO-mediated anti-tumor response. Consistently, we found an increase in CD8^+^ T cells and a decrease in CD11b^+^Ly6G^−^ immunosuppressive mononuclear population in the tumor microenvironment of *Il1rn* KO-derived tumors. To monitor the inhibitory effects of IL-1Ra on immune cells, we utilized a luciferase-based reporter CD4^+^ T cell line and splenocytes, which were derived from transgenic mice expressing ovalbumin-specific T cell receptors in CD8^+^ T cells, and mice immunized with ovalbumin. We showed that IL-1Ra suppressed T cell receptor signaling and inhibited antigen-specific interferon-γ (IFN-γ) secretion and cytolytic activity in splenocytes.

**Conclusions:**

Our findings illustrate the immunosuppressive properties of the natural anti-inflammatory cytokine IL-1Ra, and provide a rationale for considering IL-1Ra-targeted therapies in the treatment of PDA.

**Supplementary Information:**

The online version contains supplementary material available at 10.1186/s13578-023-01090-8.

## Background

Over the past several decades, the five-year relative survival rate of pancreatic ductal adenocarcinoma (PDA) has improved significantly, increasing from a mere 2.5% to a more hopeful 8.5%; however, despite this progress, PDA remains an oncological challenge [1]. While early detection of resectable tumors and subsequent adjuvant chemotherapy can increase the survival rate to a remarkable 30%, most PDA cases are diagnosed at late stages, presenting with local advanced or metastatic conditions that are associated with a grim outcome [2]. To date, immunotherapies aimed at PD-L1 and CTLA-4 have shown efficacy against various solid tumors, but neither monotherapy nor combinational treatment has produced promising results in PDA [3–5]. The lack of response to current immunotherapies suggests the existence of a unique immunosuppressive tumor microenvironment (TME) in PDA. It has been shown that numerous types of immunosuppressive cells, including tumor-associated macrophages and myeloid-derived suppressor cells, can infiltrate at an early stage, even in preinvasive lesions, whereas effector T cells are notably scarce [6]. Consequently, the cellular immunity against tumors may not receive adequate training and development. In addition, gene expression analysis has shown that high cytolytic T cell activity in PDA correlates with increased expression in many immune checkpoint genes, except for PD-L1 expression, which is notably low [7]. Hence, targeting immune-modulating genes closely associated with PDA represents a promising therapeutic direction.

Nearly all PDA harbor active mutations in the *KRAS* gene [8], but *KRAS* mutations alone are insufficient for the development of PDA without the assistance of pancreatitis-induced inflammation [9, 10]. Many pathways can regulate the inflammatory program in PDA, with interleukin (IL)-1 playing a critical role [11, 12]. Intriguingly, IL-1 receptor antagonist (IL-1Ra, gene name: *IL1RN*), another member of the IL-1 family, can inhibit IL-1 signaling [13], and patients with PDA exhibit serum concentrations of IL-1Ra over 100-fold greater than those of IL-1 agonists [14, 15]. Moreover, chemotherapy can induce serum IL-1Ra concentrations that are even 1000-fold greater [16]. These findings raise questions about the role of IL-1Ra in PDA. On the one hand, increased levels of IL-1Ra may act to prevent inflammation-promoted tumor progression. On the other hand, the anti-inflammatory properties of IL-1Ra may suppress anti-tumor immunogenic responses, as it can inhibit production of effector cytokines in CD4 T cells [17] and antigen-specific T cell responses in mice that have been immunized with tumor neoantigens [18]. Notably, single-cell RNA sequencing in mice containing pancreas-specific *Kras* mutant has shown that IL-1Ra is induced during acinar metaplasia [19], which may contribute to the early onset of an immunosuppressive TME in PDA [6]. As a result, the precise role of IL-1Ra in PDA progression remains unclear.

In this study, we have validated the elevated expression of IL-1Ra in human PDA. We compared the tumor growth in immune-competent and immune-deficient mice and found that the disruption of *Il1rn* gene resulted in severe suppression in the former. Analyzing the *Il1rn* KO-derived tumors collected from immune-competent mice showed an increase in CD8^+^ T cells and a decrease in immunosuppressive mononuclear population. Furthermore, by utilizing splenocytes to mimic complex interplay of immune cells, we showed that IL-1Ra inhibited T cell activation and cytolytic activity. Our findings support a model that tumor cell-derived IL-1Ra is involved in immunosuppression and is a potential therapeutic target of PDA.

## Results

### Increased IL-1Ra expression is associated with malignant progression in PDA

**Fig. 1:**
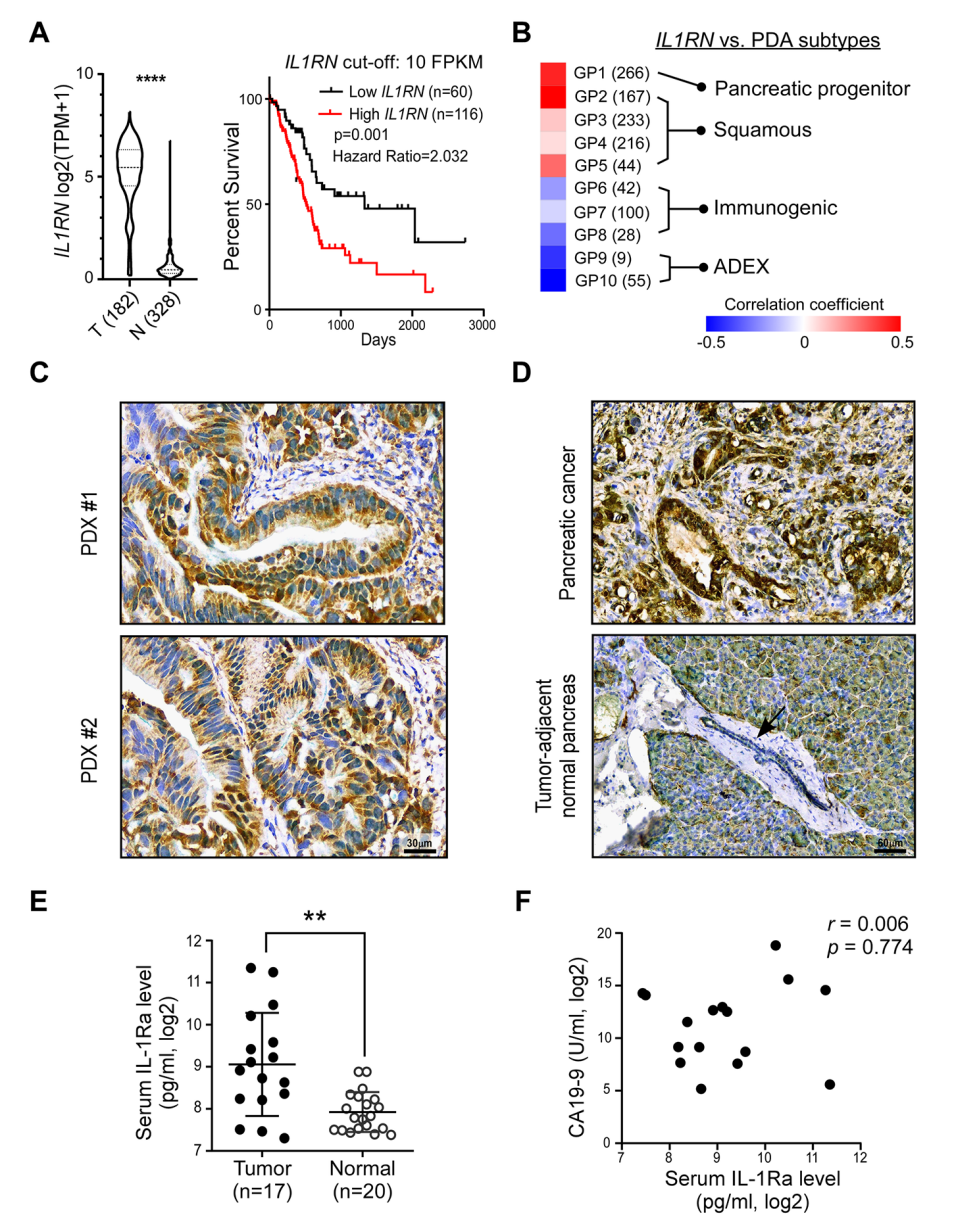
Increased expression of interleukin-1 receptor antagonist is associated with malignant progression of PDA. **(A)** Left: Comparison of *IL1RN* mRNA level between PDA (T) and healthy pancreas (N). Expression data were analyzed from the PAAD dataset of The Cancer Genome Atlas (TCGA) (n = 182) and the normal pancreas dataset of GTExPortal (n = 328). TPM: transcript per million. Student’s t-test was performed. Right: Kaplan-Meier survival analysis between two groups of patients by a cut-off value of *IL1RN* mRNA expression (low IL1RN vs. high IL1RN). FPKM: fragments per kilobase of exons per million mapped fragments. Data were analyzed by Human Protein Atlas website. **(B)** Correlation analyses between *IL1RN* mRNA level and ten gene programs (GP1-GP10), which were assigned into four subtypes of PDA. Each gene program consists of a different number of signature genes (labeled in parenthesis). Correlation coefficients are visualized by a heat map. **(C)** Immunohistochemical (IHC) analysis using an antibody against IL-1Ra protein (gene name: *IL1RN*) (brown). Patient-derived xenografts (PDX#1-#2) were derived from patients with PDA. Scale: 30 μm. **(D)** IHC analyses of IL-1Ra (brown) and FOXP3 (green) using clinical tissues directly from a PDA patient (upper panel). An identical procedure was performed in tumor-adjacent normal pancreas from the same patient, showing epithelial cells of a pancreatic duct (arrow) (lower panel). Scale: 60 μm. **(E)** Monitoring human serum IL-1Ra levels by ELISA. Sera were collected from PDA patients (Tumor, n = 17) and healthy donors (Normal, n = 20) at Taipei Medical University Hospital (Taipei, Taiwan). Student’s t-test was performed. **(F)** Correlation analysis of serum IL-1Ra and CA19-9 in the PDA patients’ sera (n = 17). *r*: Pearson’s correlation coefficient. * p < 0.05. ** p < 0.01. **** p < 0.0001

The clinical relevance of IL-1Ra in PDA was evaluated by assessing its transcriptional messenger (m)RNA levels in the TCGA database. We found that *IL1RN* mRNA was significantly elevated in PDA (T) compared to the normal pancreas (N) (Fig. 1A, left), with this increased *IL1RN* expression being correlated with poor survival (Fig. 1A, right) and advanced stages (Additional file 1: Fig. S1). No significant correlation was found between *IL1RN* and the two IL-1 agonists (Additional file 1: Fig. S2). Transcriptional network analysis previously identified ten gene programs that categorize pancreatic cancer into four subtypes [20]. Our findings showed that *IL1RN* was positively correlated with gene programs linked to both squamous and pancreatic progenitor subtypes, while negatively correlated with the immunogenic and ADEX groups (Fig. 1B). These results suggest that IL-1Ra expression is increased in certain subtypes of PDA.

Following analysis of *IL1RN* mRNA levels, we examined IL-1Ra protein contents in different types of clinical samples. First, using PDXs collected from successful growth of the human PDA in nude mice, enriched IL-1Ra protein (brown) was detected in tumor cells but not in the stromal regions (#1&#2, Fig. 1C). Next, IHC analysis of pathological tissues obtained directly from human PDA revealed a consistent increase in IL-1Ra protein expression (upper panel), while no signal was detected in the tumor-adjacent normal ductal area (arrow, lower panel, Fig. 1D). Furthermore, serum IL-1Ra levels were found to be significantly higher in patients with PDA compared to healthy individuals (Fig. 1E). However, there was no correlation between serum IL-1Ra and the established tumor marker, CA19-9 (Fig. 1F). These findings support the conclusion that increased expression of tumor cell-derived IL-1Ra accounts for the increased IL-1Ra levels observed in the TME and sera.

### ***Il1rn*** disruption does not sensitize the PDA cell line to IL-1β-dependent proliferation effect

**Fig. 2:**
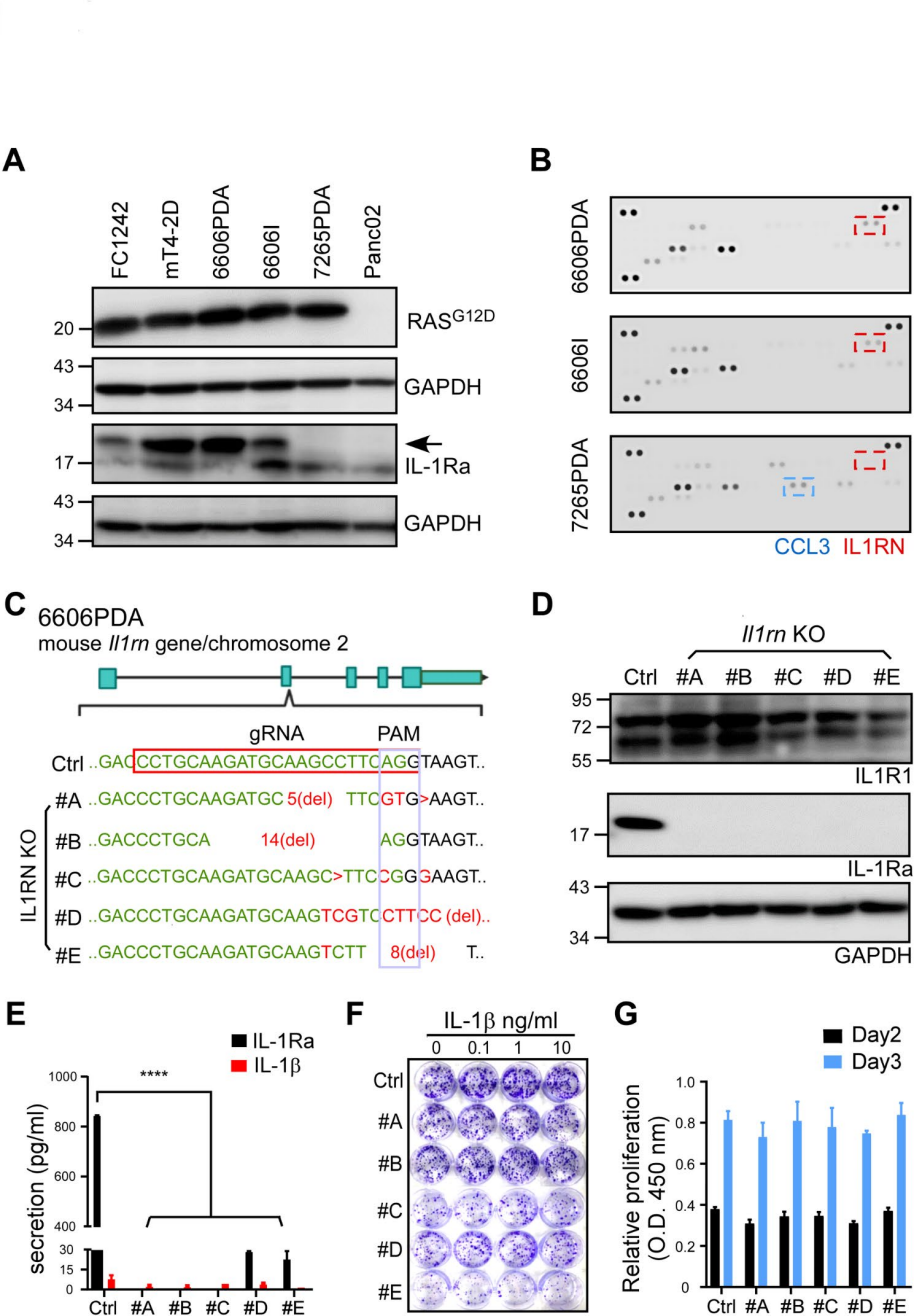
Characterization of mouse PDA with *Il1rn* knockout (KO). **(A)** Western blot analysis with a panel of mouse PDA cell lines. Specific antibodies recognizing KRAS^G12D^ mutant and IL-1Ra were used. GAPDH serves as loading control. **(B)** Comparison of cytokine and chemokine profiles in mouse PDA cell lines. Conditioned media were applied to a membrane coated with multiple antibodies against cytokines and chemokines. IL-1Ra (red) and CCL3 (blue) are labeled with dashed boxes. **(C)** Sequence analysis of the 6606PDA-derived *Il1rn* KO clones (#A~#E) using a CRISPR-Cas9 system. Exons of mouse *Il1rn* gene are labeled green, and the guide (g)RNA sequence in exon 2 is enclosed by a red box. Protospacer adjacent motif (PAM) is enclosed by a gray box. Deleted nucleotides are labeled as “del” or “>”. Nucleotide substitutions are labeled red. Cells transfected with the CRISPR-Cas9 vector without the gRNA serve as a reference (Ctrl). **(D)** Western blot analysis of IL-1 receptor (IL1R1) and IL-1Ra expression in the 6606PDA-derived clones (Ctrl & #A~#E). **(E)** Analysis of IL-1Ra and IL-1β in the conditioned media collected from 6606PDA-derived clones by an ELISA (n = 3). **(F)** Monitoring the effect of IL-1β (0–10 ng/ml) on 6606PDA-derived clones by a colony formation assay. **(G)** Proliferation analysis of the 6606PDA-derived clones (Ctrl & #A~#E) by CCK-8 assay (n = 6). Student’s t-test: ** p < 0.01. *** p < 0.001

Since IL-1Ra expression was clinically relevant in PDA, we sought to investigate the function of tumor cell-derived IL-1Ra. Given the prevalence of *KRAS* mutations in pancreatic cancer, we analyzed a panel of cell lines possessing either *Kras*^G12D^/*TP53* double mutants (FC1242 and mT42D) [21, 22] or *Kras*^G12D^ mutation alone (6606PDA, 6606I, and 7265PDA) [23]. Our results suggested that tumorigenic *Kras*^G12D^ cell lines (FC1242, mT42D, 6606PDA, and 6606I) expressed IL-1Ra, while non-tumorigenic 7265PDA [23] and Panc02 lines, which lacked *Kras* mutations [24], did not express IL-1Ra (Fig. 2A). Analysis of the conditioned media from the three *Kras* mutant cell lines showed that only tumorigenic cell lines secreted IL-1Ra (red boxes), while the non-tumorigenic 7265PDA did not (Fig. 2B). Although CCL3 (blue box) was linked to anti-tumor effects [25], its role in PDA is unclear.

Using a CRISPR-Cas9 system, we generated several *Il1rn* KO clones in 6606PDA (Fig. 2C) and confirmed their absence of IL-1Ra expression (Ctrl vs. #A-#E, Fig. 2D). The low levels of IL-1Ra secretion in the conditioned media (C.M.) from the KO clones compared to the Ctrl also confirmed IL-1Ra disruption (Fig. 2E). Since IL-1β has been shown to exhibit both pro-tumor [12] and anti-tumor effects [26–29], we examined whether the proliferation activities in response to IL-1β were influenced by *Il1rn* KO. However, even at a dosage of 10 ng/mL, IL-1β did not affect the colony formation activities of the Ctrl and KO clones (Fig. 2F). Furthermore, using CCK-8 proliferation assay did not observe differences among the clones (Fig. 2G). In summary, we established *Il1rn* KO clones that were unresponsive to IL-1 signaling.

### Functional immunity promotes anti-tumor effect against tumors derived from ***Il1rn*** KO cells

**Fig. 3:**
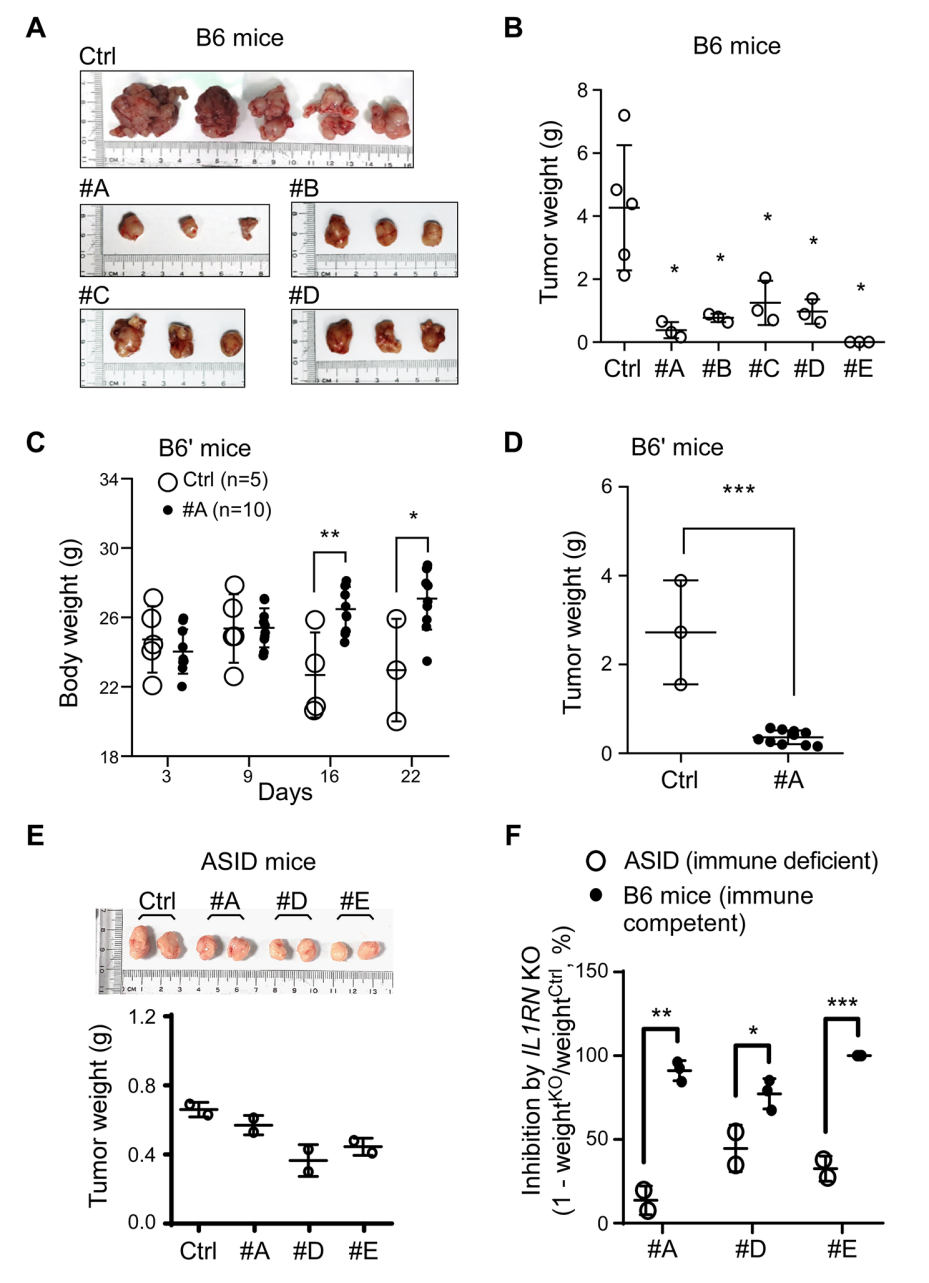
Enhanced anti-tumor activity by the functional immune system on *Il1rn* knockout (KO)-derived tumors. **(A)** Images of tumors collected from the pancreas of immune-competent C57BL/6JNarl (B6) mice after orthotopic injection with 6606PDA-derived cell lines. No tumors were detected in mice injected with #E clone. (Ctrl: n = 5; KO clones: n = 3) **(B)** Statistical analysis of tumor weights from panel A. **(C)** Comparison of body weights between Ctrl and #A clone in another set of orthotopic injected C57BL/6NCrlBltw (B6’) mice (Ctrl: n = 5; #A: n = 10). Two mice injected with Ctrl cells died during the experiment. **(D)** Statistical analysis of tumor weights from panel C. **(E)** Analysis of tumor growth in advanced severe immune deficient (ASID) mice. Images of tumors collected from the ASID mice orthotopically injected with tumor cells (upper panel). Tumor weights were measured and compared (lower panel). **(F)** Comparison of IL1RN KO-dependent inhibitory effects between ASID and B6 mice. Data in panel B and panel E were analyzed following the formula: 1-weight^KO^/weight^Ctrl^. Ctrl: parental 6606PDA cells. #A~#E: IL1RN-KO 6606PDA cells. Student’s t-test: * p < 0.05. ** p < 0.01. *** p < 0.001

To explore the biological function of tumor cell-derived IL-1Ra, we conducted an orthotopic injection procedure with immune-competent C57BL/6JNarl mice (designated B6), using the Ctrl and *Il1rn* KO clones. Our results showed a significant reduction of tumor growth in all KO clones, with clone #E exhibiting no detectable tumors (Fig. 3A and B). This indicates a role for IL-1Ra in maintaining tumor growth. Additionally, we selected clone #A and the Ctrl for comparison in another immune-competent strain (C57BL/6NCrlBltw, labeled B6’). We observed a significant weight loss and poor survival rate only in mice injected with Ctrl, while most mice injected with clone #A exhibited a steady increase in body weight (Fig. 3C). Once again, disruption of IL-1Ra led to inhibited tumor growth in the B6’ strain (#A, Fig. 3D). To investigate the impact of functional immunity, we performed experiments using immune-deficient ASID mice. In contrast to the immune-competent mice, we observed a marked growth of clone #E, and all the KO clones showed comparable tumor sizes with the Ctrl (Fig. 3E). Statistical analysis confirmed a significantly stronger anti-tumor effect of *Il1rn* KO in immune-competent mice than in ASID mice (Fig. 3F). These results suggest that the anti-tumor effect of *Il1rn* KO largely depends on the presence of an intact immune system.

### ***Il1rn*** KO-derived tumors exhibit reduced immunosuppressive properties in the TME

**Fig. 4:**
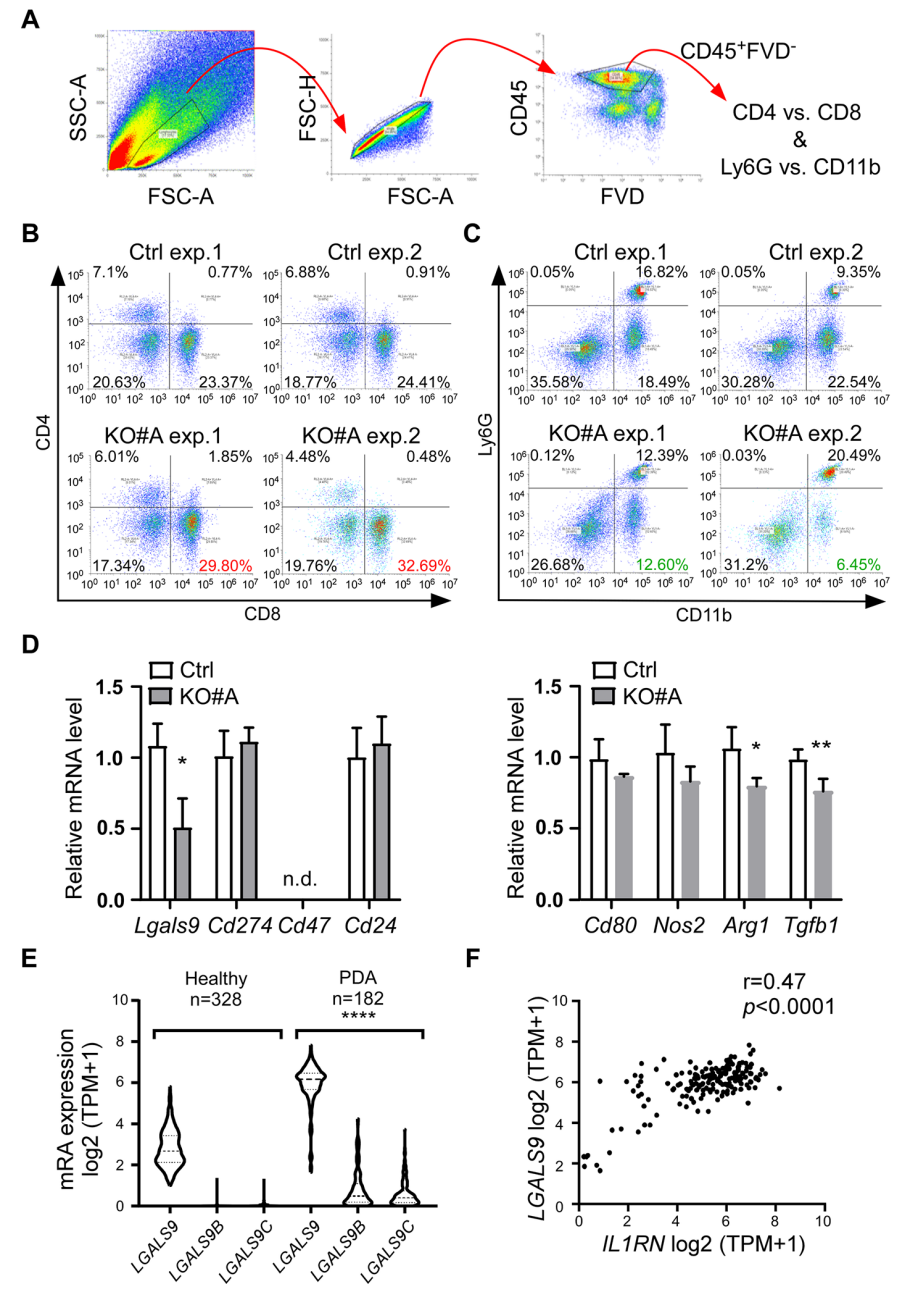
Perturbation of tumor microenvironment in *Il1rn* knockout (KO)-derived tumors. **(A)** Immune profile analysis in the tumor microenvironment (TME). Viable CD45-positive populations (CD45^+^FVD^−^) were collected for further analysis by several markers (CD4, CD8, Ly6G, and CD11b). FVD: a fixable viability dye to monitor cell viability. **(B** & **C)** CD45^+^FVD^−^ population was analyzed by two combinations (CD4 vs. CD8, panel B; Ly6G vs. CD11b, panel C). Tumors collected from mice injected with either parental 6606PDA cells (Ctrl) or the *Il1rn* knockout clone (KO#A) were assigned to two groups (exp.1 & exp.2, n = 2–3 tumors per set). Increased CD8^+^ populations in clone #A-derived tumors were labeled red while reduced CD11b^+^Ly6G^−^ were green. **(D)** Relative mRNA levels in the Ctrl and *Il1rn* KO (KO#A) tumors. Selected mouse genes were analyzed by qPCR. n = 3. Student’s t-test was performed. **(E)** Comparison of human galectin-9 paralogs (*LGALS9*, *LGALS9B*, and *LGALS9C*) mRNA expressions between normal pancreas (Healthy, n = 328) and PDA (n = 182). Student’s t-test was performed. **(F)** Correlation analysis of human IL-1Ra (*IL1RN)* and galectin-9 (*LGALS9)* mRNA expression using the PDA dataset from TCGA (n = 182). r: correlation coefficient. TPM: transcript per million. * p < 0.05. ** p < 0.01. **** p < 0.0001

The anti-tumor effect of *Il1rn* KO has promoted us to monitor the immune profile of T cells and myeloid (CD11b^+^) populations in the TME (Fig. 4A). We observed that CD8^+^ T cells were increased by approximately 6% in *Il1rn* KO-derived tumors compared to tumors from the Ctrl clone (Ctrl vs. KO#A, Fig. 4B). In contrast, the population of immunosuppressive mononuclear cells (CD11b^+^Ly6G^-^) [30, 31] was reduced by roughly 10% (Ctrl vs. KO#A, Fig. 4C). Furthermore, we examined the expression of genes related to immune checkpoints [32] and the polarization of macrophages [33], and found that galectin-9 (*Lgals9*) and M2-macrophage (*Arg1* and *Tgfb1*) were reduced in the *Il1rn* KO-derived tumors (KO#A, Fig. 4D). Galectin-9 has been involved in T cell death [34] and immunosuppressive macrophages [35]. By analyzing TCGA database, we found that all the *LGALS9* paralogues, including two *LGALS9*–like genes [36], were increased in PDA, and a tight correlation existed between *IL1RN* and galectin-9 (Fig. 4F). These results are consistent with the increased CD8^+^ T cells and suppressed CD11b^+^Ly6G^-^ populations in the *Il1rn* KO-derived tumors. In summary, our results support that tumor cell-derived IL-1Ra contributes to the immunosuppressive TME.

### T cell activation by dendritic cells is suppressed by IL-1Ra

**Fig. 5:**
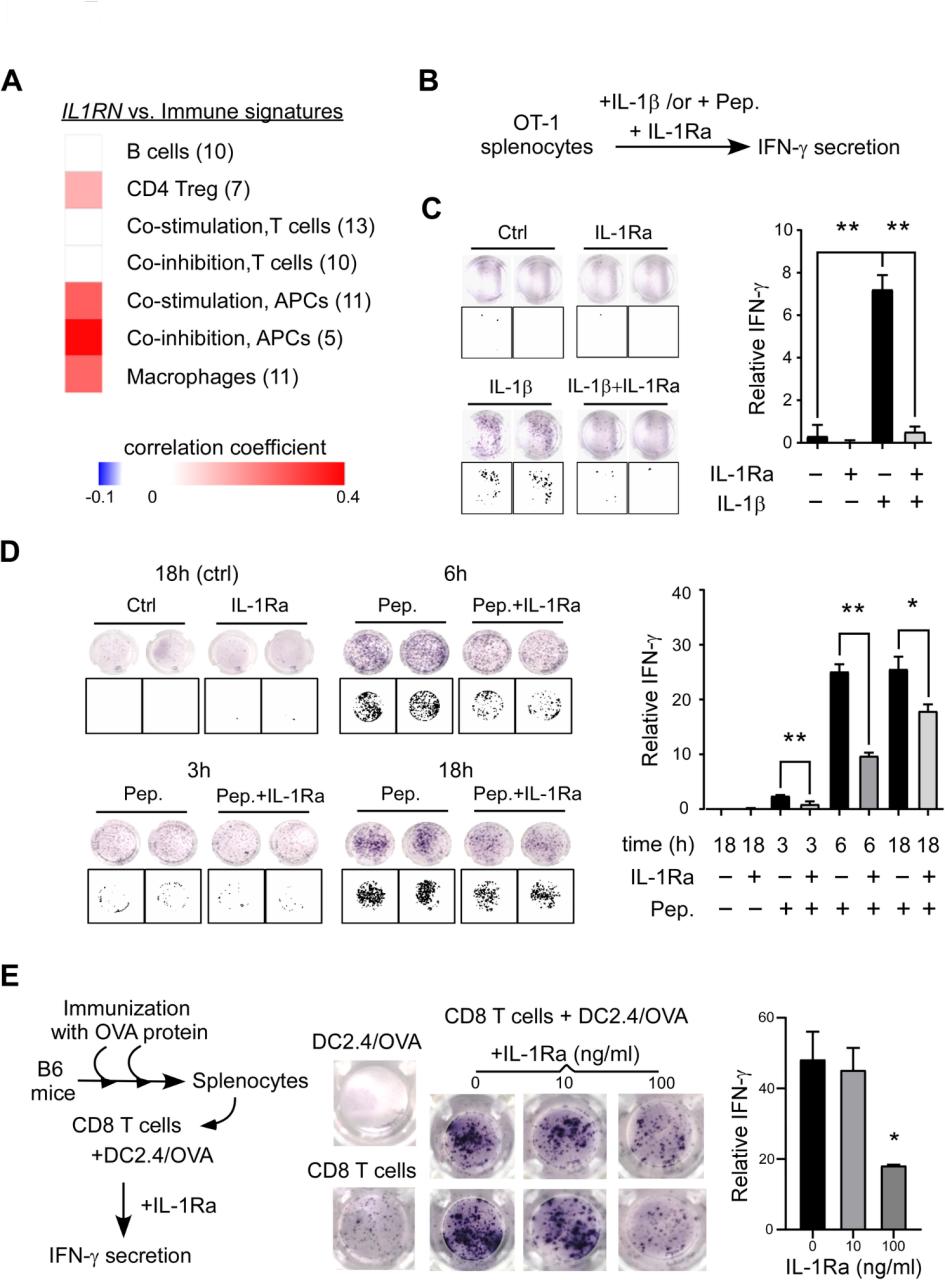
Inhibition of antigen-specific activation of T cells by IL-1Ra. **(A)** Correlation analyses between *IL1RN* mRNA level and seven immunogenic gene programs associated with PDA. Each immunogenic program consists of different number of signature genes (labeled in parenthesis). Correlation coefficients are labeled under the gene programs. Treg: regulatory T cells. APCs: antigen-presenting cells. **(B)** The flowchart of testing inhibitory effects of IL-1Ra on IL-1β or antigen (Pep.)-induced interferon (IFN)-γ secretion by the splenocytes collected from OT-1 mice (OT-1 splenocytes). Pep.: an OT-1 peptide (SIYRYGGL) specific for activating CD8^+^ T cells of OT-1 mice. **(C)** IL-1Ra protein inhibits IL-1β-induced IFN-γ secretion by OT-1 splenocytes. OT-1 splenocytes were treated with the combination of recombinant IL-1Ra (100 ng/ml) and IL-1β (1 ng/ml) for 18 h. IFN-γ-positive colonies were monitored by an ELISPOT assay and images were processed by ImageJ software (boxes). Student’s t-test was performed (right). **(D)** IL-1Ra inhibits antigen-induced IFN-γ secretion. IFN-γ secretion at different time points (3 h, 6 h, and 18 h) in OT-1 splenocytes following a combination of 0.1 ng/ml OT-1 peptide (Pep.) and 100 ng/ml IL-1Ra treatment. OT-1 splenocytes without OT-1 peptide treatment served as negative control (no Pep.). Right: ELISPOT images were processed and quantified. Student’s t-test was performed. **(E)** IL-1Ra inhibits CD8^+^ T cell activation by dendritic cells. Left: CD8^+^ T cells were enriched from ovalbumin (OVA)-immunized mice and incubated with OVA-pulsed DC2.4 dendritic cells (DC2.4/OVA), followed by detection of IFN-γ secretion. Middle: IFN-γ secretion in a mixture of CD8^+^-T cell and DC2.4/OVA in response to different concentration of IL-1Ra. Immune cells without IL-1Ra treatment serve as a negative control. Right: ELISPOT images were processed and quantified. Student’s t-test was performed. * p < 0.05. ** p < 0.01. *** p < 0.001. **** p < 0.0001

The interactions between antigen-presenting cells (APCs) and T cells are crucial for activating cytotoxic T lymphocytes (CTLs) in anti-cancer response [37]. The underlying mechanism may rely on the APC-secreted IL-1, which activates the IL-1 receptor (IL1R1) signaling in CD4^+^ T cells and promotes their production of effector cytokines (e.g., IFN-γ) [17]. Using gene programs associated with immune populations in PDA [20], we found a positive correlation between *IL1RN* mRNA and gene programs related to CD4^+^ Treg, APCs, and macrophages (Fig. 5A). Moreover, *IL1RN* was negatively correlated with genes involved in cross-presentation (e.g., XCR1) [38] (Additional file 1: Fig. S3). These findings suggest a regulatory role of IL-1Ra in APC-mediated T cell activation.

To address this issue, we replicated the APC-T cell interaction using the OT-1 splenocytes (Fig. 5B). These splenocytes were derived from OT-1 transgenic mice whose CD8^+^ T cells expressed the ovalbumin (OVA) 257–264 epitope-specific T cell receptors [39]. Adding IL-1β in the OT-1 splenocytes can induce IFN-γ secretion, which was suppressed by IL-1Ra, demonstrating the functional presence of IL-1 signaling (Fig. 5C). Upon exposing the system to its cognate peptide (Pep., OVA 257–264), IFN-γ secretion was increased in a time-dependent manner, again suppressed by the addition of IL-1Ra (Pep.+IL-1Ra, Fig. 5D). To confirm the antigen specificity of OT-1 splenocytes, we demonstrated that IFN-γ secretion was only induced by the cognate OVA 257–264 peptide and not by an unrelated peptide (Additional file 1: Fig. S4). These results indicate that IL-1Ra inhibits the positive function of IL-1 in APC-mediated T cell activation.

To further test the effect of IL-1Ra, we reconstituted the system utilizing solely CD8^+^ T cells and dendritic cells. CD8^+^ T cells were enriched from B6 mice immunized with full-length OVA and incubated with a syngeneic dendritic cell line pulsed with OVA (DC2.4/OVA, Fig. 5E). Our results demonstrated that IL-1Ra suppresses the secretion of IFN-γ from the mixture of CD8^+^ T cells and DC2.4/OVA (Fig. 5E), supporting a positive role of IL-1 in APC-medicated restimulation of memory CD8^+^ T cells. In conclusion, our findings support the immunosuppressive role of IL-1Ra.

### IL-1Ra suppresses the T cell activation signaling and cytolytic activity of OT-1 splenocytes

**Fig. 6:**
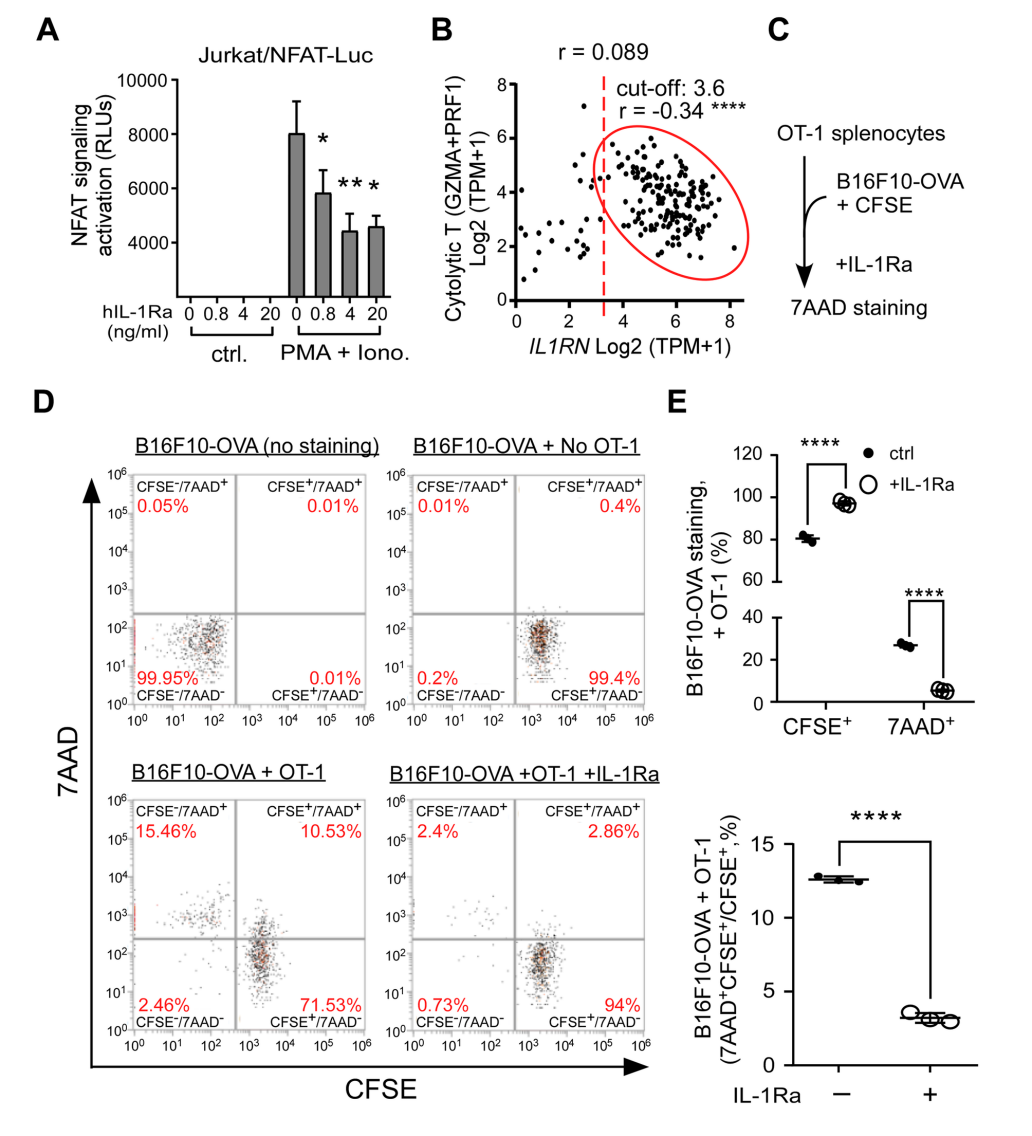
Inhibitory effects of IL-1Ra on T cell signaling and cytotoxic activity of OT-1 splenocytes. **(A)** Inhibition of IL-1Ra on a CD4 T cell line (Jurkat/NFAT-Luc) containing a luciferase reporter in response to NFAT signaling. Jurkat/NFAT-Luc cells were treated with PMA (20 ng/ml) and ionomycin (iono., 1 mg/ml), and relative luciferase light units (RLUs) were monitored in response to a dosage of IL-1Ra. Student’s t-test was performed. **(B)** Correlation analysis between *IL1RN* mRNA level and a cytolytic T cell index consisting of an average of *GZMA* and *PRF1* mRNA level. *r*: Pearson’s correlation coefficient. **(C)** A flowchart of testing the inhibitory effect of IL-1Ra on cytolytic activity of OT-1 splenocytes. A melanoma cell line expressing full-length ovalbumin (B16F10-OVA) was labeled with CFSE and then incubated with OT-1 splenocytes. The effect of IL-1Ra was monitored by measuring the level of 7AAD staining. **(D)** Monitoring 7AAD and CFSE staining of B16F10-OVA cells in different conditions (No OT-1, +OT-1, +OT-1 + IL-1Ra) by flow cytometry. Percentages of cell populations were labeled red. **(E)** Inhibitory effects of IL-1Ra on cytolytic activities of OT-1 splenocytes. Changes of CFSE-positive (CFSE^+^) or 7AAD-positive (7AAD^+^) B16F10-OVA cells incubated with OT-1 splenocytes in response to IL-1Ra were analyzed (upper panel). The ratio of double-positive B16F10-OVA cells (7AAD^+^CFSE^+^) over CFSE^+^ ones was compared in response to IL-1Ra (lower panel). Student’s t-test was performed. * p < 0.05. **** p < 0.0001

Our results have suggested an inhibitory role of IL-1Ra in APC-mediated T cell activation, we further asked whether IL-1Ra can influence T cell activation signaling by employing a luciferase reporter CD4^+^ T cell line (Jurkat/NFAT-Luc), since IL-1 signaling in CD4^+^ T cells is crucial for effector cytokine production [17]. Indeed, elevated IL-1Ra levels suppressed the NFAT signaling, which was stimulated by a combination of ionomycin (Iono.) and PMA [40] in the Jurkat/NFAT-Luc cell line (Fig. 6A). Gene expression of granzyme A (*GZMA*) and perforin (*PRF1*) has been reported to serve as a cytolytic index of T cells [7]. When analyzing the correlation between the cytolytic index and *IL1RN*, we observed a biphasic pattern in PDA populations (Fig. 6B). When the *IL1RN* expression is above a threshold, the cytolytic activity decreases inversely with *IL1RN* (red circle, Fig. 6B). These results support the immunosuppression hypothesis that IL-1Ra inhibits T cell function.

To test this hypothesis, we analyzed the cytolytic activity of OT-1 splenocytes against a melanoma cell line expressing the full-length OVA protein (B16F10-OVA), which was shown to be selectively targeted by OT-1 CD8^+^ T cells [41] (Fig. 6C). Perforin/granzyme-mediated cell death, the major killing mechanism employed by CTLs [42], was quantified by staining with a DNA intercalating dye (7AAD) and a protein-conjugating dye (CFSE), which labels cells with permeable membranes and monitors the protein level in individual cells (Fig. 6C). We also confirmed that the CFSE-stained B16F10-OVA cells could be clearly distinguished from OT-1 splenocytes using flow cytometry analysis (Additional file 1: Fig. S5).

Compared to B16F10-OVA cells alone (No OT-1), treatment with the OT-1 splenocytes increased the loss of protein content (OT-1), while co-treatment with IL-1Ra inhibited the effect (OT-1 + IL-1Ra, Fig. 6D). CFSE-based assay was shown to be as sensitive as the conventional ^51^Cr release assay in quantifying cytotoxic T-cell responses [43]. Our results showed that IL-1Ra protected the protein loss from ca. 80% back to 97% (CFSE^+^) and suppressed membrane poration from ca. 25–5% (7AAD^+^, upper panel, Fig. 6E). IL-1Ra may protect tumor cells from membrane poration prior granzyme-mediated protein degradation, as evidenced by the significant decrease of 7AAD^+^ pool in CFSE^+^ populations (from ca. 13–3%, lower panel, Fig. 6E). Overall, our results support the role of IL-1Ra in immunosuppression and as a therapeutic target.

## Discussion

The anti-cancer response following APC-T cell interaction involves the APC-secreted IL-1, which activates IL1R1 signaling in CD4^+^ T cells and promotes production of many effector cytokines [17]. Therefore, our results showing that IL-1Ra compromises the NFAT signaling in the APC-free, chemically defined activation of Jurkat/NFAT-Luc system (Fig. 6A), suggest an autonomous IL-1-IL1R1 axis in the T cell activation. It should be noted that, although the stimulated CD4^+^ T cells respond similarly to either IL-1α or IL-1β, the APCs preferentially utilize IL-1β after interaction with T cells [17]. One explanation for this preference is that APCs can respond to multiple types of damage molecules in the local environment by utilizing the sophisticated inflammasome machinery, which is specialized to produce mature IL-1β, not IL-1α, to broadcasts the alarming messages [44]. Importantly, in addition to APCs, human CD4^+^ T cells isolated from healthy donors have also been shown to possess inflammasome, with secreted IL-1β serving as an autocrine factor that promotes T helper 1 differentiation [45]. Moreover, previous studies have also demonstrated increased expression and secretion of IL-1β in activated Jurkat cells induced by phytohemagglutinin [46, 47]. In summary, IL-1Ra can suppress NFAT signaling by blocking both the APC-secreted IL-1β and the autocrine effect of T cell-derived IL-1β.

NFAT signaling is critical for the activated CD4^+^ and CD8^+^ T cells (the CTLs), inducing genes like IFN-γ and granzyme [48, 49]. Following TCR signaling, the elevated intracellular calcium level is a critical step to activate NFAT transcription factor for regulating the polarization program in T helper cells, and one important player in regulating the calcium influx is the calcium release-activated calcium channel [50]. However, the transient receptor potential ankyrin-1 (TRPA1), a calcium permeable ion channel, was also shown to be a functional regulator in CD4^+^ T cell [51]. Importantly, other studies have reported that IL-1β can enhance TRPA1-dependent calcium influx and increase the expression of TRAP1 [52, 53]. It is possible that IL-1β regulates many other types of calcium channels (e.g., NMDA receptor) [54, 55], and their roles in the activity of NFAT signaling and T cell functions require further investigation. In addition to regulating intracellular calcium level via sensitizing the activating threshold of NFAT signaling, IL-1-IL1R1 signaling also plays a key role in maintaining stabilities of the cytokine transcripts in activated T cells [17]. Consistent with this, it was shown that the intracellular IL-1Ra can attenuate the mRNA stability of IL-1 induced genes [56], likely due to P2X7 receptor-mediated secretion of IL-1Ra [57]. In summary, these findings support the notion that IL-1Ra exerts its effects through multiple pathways, such as blocking upstream calcium influx or downstream RNA stability, to suppress NFAT signaling and anti-tumor activity.

Our results validated the inhibitory role of IL-1Ra in APC-mediated T cell activation using the OT-1 splenocytes and the constituted system containing CD8^+^ T cells enriched from the mice immunized with OVA and OVA-pulsed DC2.4 dendritic cells (Fig. 5). Since the CD8^+^ T cells used in these systems already possess OVA-specific TCR, our results suggest that IL-1-IL1R1 signaling, in conjunction with TCR signaling, promotes the activation of antigen-experienced (primed) CD8^+^ T cells. In isolated human naïve CD8^+^ T cells, it has been shown that only IL-1β, as compared to other proinflammatory cytokines such as IL-6 and TNF, has the capability to induce IFN-γ induction in the APC-free, antibody-defined activation system [58]. Hence, IL-1Ra may suppress immunogenic anti-tumor response at multiple stages, including the initial priming of naïve CD8^+^ T cells and the restimulation of memory ones. Consistent with this idea, despite the ability of many clinically used chemotherapeutic drugs to directly damage DNA and lead to cell death in vitro, their efficacy in vivo is significantly dependent on APC-secreted IL-1β [58]. Thus, it is possible that the aberrant expression of IL-1Ra in cancer cells inhibits the anti-tumor role of IL-1β in APC-mediated T cell activation, thereby contributing to a subtype of gemcitabine, or other types of chemotherapies, resistant PDA.

In addition to our immunosuppression model, alternative mechanisms for the tumor-promoting role of IL-1R have been suggested. In a PTEN null mouse prostate model, CD11b^+^Gr-1^+^ myeloid cells were found to play a crucial role in suppressing oncogene-induced senescence through IL-1Ra-mediated paracrine [59]. IL-1α signaling is responsible for senescence and, unlike IL-1β, is a cell surface-bound or intracellular protein [60]. It is possible that cancer cells and myeloid cells express IL-1Ra in PDA to effectively counteract IL-1α-mediated senescence signaling. Furthermore, IL-1Ra was found to stimulate proliferation by directly activating mitogenic signaling (e.g., ERK and AKT) [27, 61]. These findings support that IL-1Ra can contribute to tumor progression through various mechanisms.

This line of thought also suggests the necessity of anti-inflammatory components in the process of tumor promotion. In a PDA mouse model, adult acinar cells have been found to be resistant to transformation by the *Kras* oncogene or a combination of *Kras* and loss of tumor suppressors (e.g., *Trp53*); however, treating caerulein induces pancreatitis and accelerates the development of PDA [9]. Therefore, inflammation has been recognized a role in tumor promotion. However, it has been shown that Toll-like receptors (TLRs) are important in caerulein-induced inflammatory response [62, 63] and most mice exhibit higher expression of IL-1Ra compared to the two IL-1 agonists when treated with stimulating ligands against different TLRs (TLR1-TLR9) [18], underscoring the important role of IL-1Ra in inflammatory programs. As discussed previously, IL-1Ra can assist tumor progression through multiple mechanisms; thus, IL-1Ra induction contributes to the tumor-promoting effect of inflammation [59, 60].

When comparing the PDA model, which is induced by a *Kras* mutant and caerulein, with the skin carcinogenesis model, where a carcinogenic agent induces *Ras* mutations (initiation phase) followed by clonal expansion by a phorbol ester (promotion phase), caerulein appears to act as a tumor-promoting agent [64]. Nevertheless, unlike in the skin carcinogenesis model where the direction from initiation to promotion phase is non-interchangeable, in the PDA model, a *Kras* mutant can occur at least one month after the cessation of caerulein-induced inflammation and still reliably develop PDA [9]. This suggests that inflammation not only serves as a tumor-promoting agent but also preconditions the cells to be immune from oncogene-induced stress, which explains why pancreatitis is a major risk factor for PDA [10]. If IL-1Ra is induced during pancreatitis, which is consistent with the early induction of IL-1Ra in preinvasive lesions [19], it can utilize its unique functions, e.g., protecting *KRAS*-induced arrest and immune surveillance, to precondition the pancreas. Intriguingly, IL-1Ra expression in response to inflammatory stimuli is less efficient in humans than in mice [18]; however, we have demonstrated elevated levels of IL-1Ra in PDA clinical samples (Fig. 1). This suggests that critical events during the inflammatory responses lead to genetic or epigenetic changes that drive IL-1Ra induction, which could provide novel diagnostic and prognostic markers for PDA.

Our findings shed light on the inhibitory effects of IL-1Ra on T cell function, which contribute to the early establishment of an immunosuppressive TME in PDA [6]. The induction of IL-1Ra in preinvasive lesions [19] may account for the inability of local immunity to engage with neoantigens and elicit antigen-specific cellular responses, leading to a limited recruitment of suppressed yet primed CD8^+^ T cells by immune checkpoint inhibitors. In addition to tumor cell-derived IL-1Ra, M2 macrophages may also provide a significant source of IL-1Ra in the TME [33]. Furthermore, previous studies have also demonstrated that mesenchymal stem cell-derived IL-1Ra is important in maintaining the M2 macrophages [65–67]. Therefore, various types cells, through both paracrine and autocrine mechanisms, can contribute to the immunosuppressive effects of IL-1Ra in the TME of PDA. Clinical responses to immune checkpoint inhibitors have shown that pancreatic and prostate cancers belong to the category of “cold” tumors, while melanomas and lung cancers are classified as “hot” tumors [68]. The presence of IL-1Ra in the TME of a mouse model of prostate cancer but not lung cancer has suggested that IL-1Ra may serve as a biomarker in certain cold tumors [27]. Further investigations are required to clarify whether targeting IL-1Ra could represent a therapeutic strategy for cold tumors or even convert them into hot tumors.

## Conclusions

**Fig. 7:**
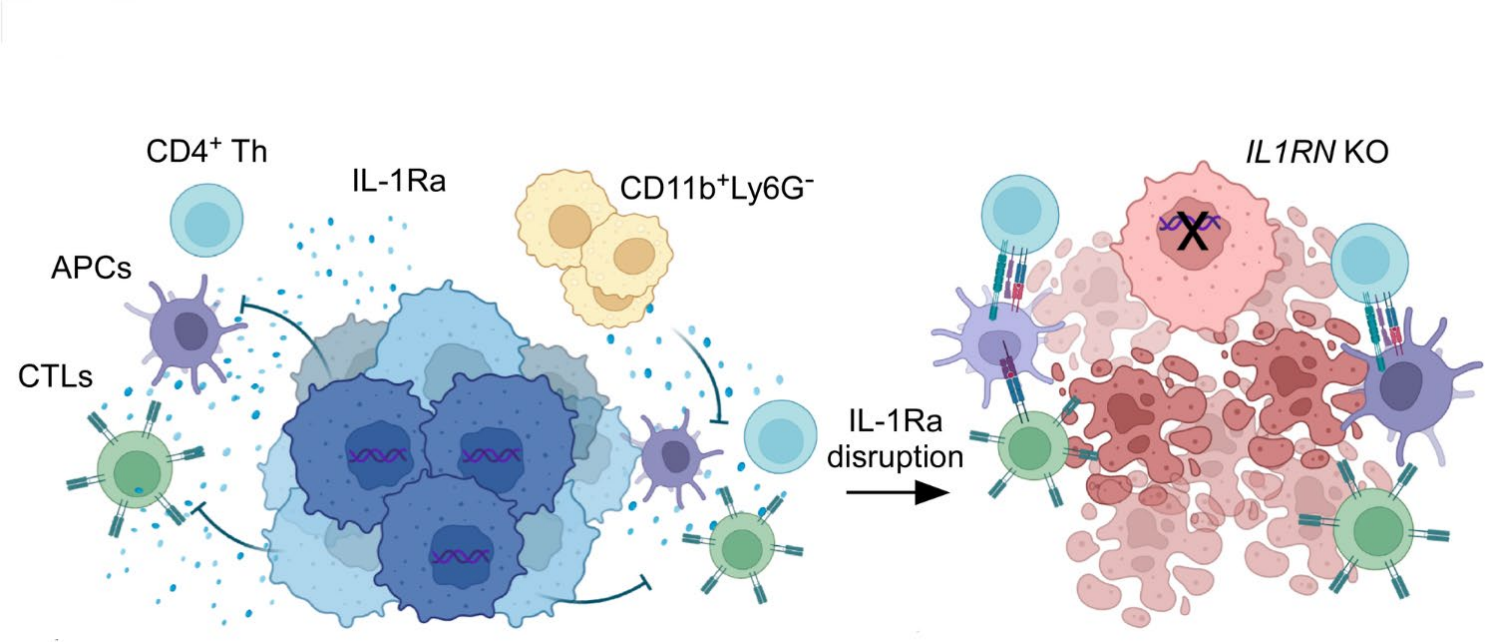
A schematic model of the tumor-promoting functions of IL-1Ra in PDA. Tumor cell-derived IL-1Ra creates an immunosuppressive tumor microenvironment (TME, blue) that inhibits immunogenic anti-cancer functions. The IL-1Ra inhibits T cells and APCs activities, and maintains immunosuppressive mononuclear cells (CD11b^+^Ly6G^−^). Interruption of IL-1Ra disrupts the immunosuppression, preventing the localization of CD11b^+^Ly6G^−^ cells and inducing inflammatory anti-tumor immune responses in the TME (red). Thus, APCs can successfully coordinate the signals from CD4^+^ T helper and lead to anti-tumor activation of CTLs. Th: T helper cells. CTLs: CD8^+^ cytotoxic T-cell lymphocytes. APCs: antigen-presenting cells. KO: gene knockout

In summary, our findings indicate a correlation between heightened levels of IL-1Ra and the malignant progression of PDA, and propose a molecular mechanism through which IL-1Ra-mediated immunosuppression fuels the development of PDA (as depicted in Fig. 7). Within the TME, IL-1Ra is capable of suppressing the immunogenic anti-tumor response by impairing the activities of APCs, CD4^+^ T cells, and CD8^+^ T cells (Fig. 7). Nevertheless, the disruption of IL-1Ra can reverse the TME toward an inflammatory, immunogenic response, leading to an anti-tumor effect. Our results suggest that tumor cell-derived IL-1Ra is a viable therapeutic target and a valuable biomarker in the management of PDA.

## Methods

### Cell lines

The mouse pancreatic cell lines (FC1242, mT4-2D, 6606PDA, 6606I, and 7265PDA) were kindly provided by Dr. David Tuveson (Cold Spring Harbor Laboratory, NY, USA; Cambridge University, UK) via a material transfer agreement, and the B16F10-OVA cell line was kindly provided by Dr. Mi-Hua Tao (Academia Sinica, Taipei, Taiwan). The DC2.4 mouse dendritic cell line was purchased from Merck Millipore (Darmstadt, Germany), and the Jurkat/NFAT-Luc cell line was purchased from InvivoGen (Hong Kong, China). The mouse pancreatic cancer cell lines were maintained in Dulbecco’s modified Eagle medium (DMEM) (Corning Inc., Corning, NY, USA). B16F10-OVA was cultured in DMEM/Nutrient Mixture F-12 (DMEM/F12) (Invitrogen, Carlsbad, CA, USA). DC2.4 cells were maintained in Roswell Park Memorial Institute (RPMI) 1640 Medium (Corning Inc.). All media were supplemented with 10% fetal bovine serum (FBS) and 1% antibiotic antimycotic solution (Corning Inc.), according to established procedures.

### Tissue samples and immunohistochemical (IHC) analysis

The use of one set of clinical patient samples, including pancreatic cancer tissues and sera, was approved by the Taipei Medical University-Joint Institutional Review Board (Taipei, Taiwan, approval no.: N202011039). The use of another set of samples for generating patient-derived xenografts (PDXs) was approved by the institutional review board of the University Medical Center Rostock (Rostock, Germany, approval nos.: II HV 43/2004, A 45/2007, and A 2018-0054) [69]. Antibodies used for IHC staining are listed in the Additional file 1: Table S1. In general, tissue sections were deparaffinized and rehydrated. Antigen retrieval was performed in boiling citrate buffer (pH 6.0) for 20 min. Endogenous peroxidase was blocked using a blocking buffer (TA00C2, BioTnA, Kaohsiung, Taiwan) and a 3% hydrogen peroxide solution. Primary antibodies were incubated at 26 °C for 1 h, followed by a horseradish peroxidase (HRP)-labeled anti-mouse/rabbit antibody (Vector Laboratories, Burlingame, CA, USA) at room temperature for 30 min. The expression level was detected by the TAlink mouse/rabbit polymer detection system (TADS03, BioTnA). All IHC slides were counterstained with hematoxylin. Images were digitized with a Motic Easyscan Digital Slide Scanner (Motic, Hong Kong, China) at 40 × (0.26 μm/pixel). The pathologic features were first microscopically examined by a veterinarian (Toson Technology, Hsinchu, Taiwan) followed by a medical pathologist.

### Western blot analysis

Cell lysates were prepared with 6× Laemmli sample buffer and Western blot was performed with specific antibodies (Additional file 1: Table S1). Images were taken with an AmershamTM Imager 600 (GE Healthcare, Chicago, IL, USA).

### Enzyme-linked immunosorbent assay (ELISA)

Mouse and human IL-1Ra/IL-1F3 (Quantikine ELISA Kit; R&D Systems, Minneapolis, MN, USA), mouse IL-1β ELISA kits (Abcam, Cambridge, UK), and human cancer antigen CA19-9 ELISA kit (Abcam) were used to detect specific cytokine secretions. The procedure was performed following the manufacturer’s protocol. The measurement was made by reading the absorbance at 450 nm on an Epoch Microplate Spectrophotometer (BioTek Instruments, Winooski, VT, USA).

### Proliferation assay

Cells (1000 cells/well) were seeded into 96-well plates and cultured for 2 ~ 3 days. A CCK-8 solution (Sigma-Aldrich) was applied to each well and incubated for 1 h. The signal was detected by an Epoch Microplate Spectrophotometer (BioTek Instruments).

### Colony-formation assay

Cells (500 cells/well) were seeded into 12-well plates and cultured for 5 ~ 7 days. Cells were incubated with different concentrations of IL-1β (0, 0.1, 1, and 10 ng/ml). Colonies were stained with crystal violet (Sigma-Aldrich) and photographed.

### Mouse cytokine array analysis

Secreted cytokines were measured using a mouse cytokine array panel A kit (R&D systems) and following the manufacturer’s protocol. Conditioned media of pancreatic cancer cell lines were collected as supernatants. The detection antibody cocktail was added to the supernatants for 1 h followed by incubation with membranes overnight at 4 °C. After washing, HRP-conjugated Streptavidin was added, and membranes were exposed with Chemi Reagent. Images were taken with an Amersham™ Imager 600 (GE Healthcare).

### Generation of IL1RN KO cell lines

Guide (g)RNA targeting mouse *IL1RN* exons 2 and 3 (NM_031167) was based on the CRISPR/CRISPR-associated protein 9 (Cas9) editing technique. One mouse IL1RN gRNA sequence was selected from a CRISPR-designed website (https://chopchop.cbu.uib.no) and cloned into pSpCas9(BB)-2 A-Puro (PX459) V2.0, a gift from Feng Zhang (Addgene plasmid no. 62,988) (Additional file 1: Table S2). Following transient transfection, 6606PDA cells were treated with puromycin (2.5 µg/ml) to select temporally puromycin-resistant clones. IL1RN KO clones were confirmed by several approaches: Western blotting for intracellular expression, ELISA for IL1RN secretion, and DNA sequencing for mutation profiles. Genomic DNA isolated from KO clones was amplified by a polymerase chain reaction using specific primers (Additional file 1: Table S2), followed by sequencing to validate the mutations.

### In vivo tumor cell xenograft assays

The syngeneic orthotopic mouse model was performed in accordance with a protocol approved by the Taipei Medical University Animal Care and Use Committee (no.: LAC-2020-0255, Taipei, Taiwan). An established procedure to conduct the syngeneic model was followed [70]; in brief, 2.5 × 10^5^ 6606PDA cells mixed with Matrigel (Corning Inc.) were orthotopically injected into the pancreas of 8-week-old male C57BL/6JNarl mice (NLAC, Taipei, Taiwan) or C57BL/6NCrlBltw (BioLASCO Taiwan Co., Ltd., Taipei, Taiwan). The subcutaneous model using mice (NOD.Cg-*Prkdc*^*scid*^*Il2rg*^*tm1Wjl*^/YckNarl) with low innate immunity and without lymphocytes and NKT cells (advanced severe immunodeficiency/ASID, NLAC, Taipei, Taiwan) was performed in accordance with the protocol approved by the Academia Sinica (no.: 19-11-1357). Mice were sacrificed around 5 weeks post injection, and tumors were collected for further analysis.

### Immune profiling analysis

Tumors were collected and incubated with Collagenase IV, DNase type I and Hyaluronidase (MedChemExpress, Monmouth Junction, NJ, USA) for 30 min at 37 °C. Single-cell suspensions were collected by filtering through a nylon mesh with a 70-µm pore size (Falcon Cell Strainers, Thermo Fisher Scientific). Splenocytes were collected by crushing spleens followed by filtering through a nylon mesh with a 100-µm pore size. After treatment with an Fc blocking reagent (Miltenyi Biotec, Bergisch Gladbach, Germany), samples were stained with fluorescent antibodies in the stain buffer (1x PBS, 2% BSA, 0.1% NaN3): anti-CD45 (Clone 30-F11), anti-CD11b (Clone M1/70) (BD Biosciences, Franklin Lakes, NJ, USA), anti-CD8a (Clone 53 − 6.7), anti-CD4 (GK1.5), anti-Ly6G (Clone 1A8) (BioLegend). Dead cells were labeled by the fixable viability dye eFluor™ 780 (Invitrogen). Before flow cytometry analysis, cells were fixed in the fixation buffer (BioLegend) and washed using FASC buffer. Data were collected by using Attune™ NxT Acoustic Focusing Cytometer (Invitrogen) and analyzed by the Floreada.io website.

### Antigen-dependent IFN-γ secretion by enzyme-linked immune-absorbent spot (ELISPOT) and cytotoxic assay

Splenocytes from OT-1 mice (10^5^ cells/well) were treated with cytokines (IL-1β and IL-1Ra) (ProSpec, Rehovot, Israel) and the OT-1 peptide (ovalbumin (OVA) 257 ~ 264 a.a. 100 ng/ml) in each well. For enriching CD8^+^ T cells that recognize ovalbumin-derived antigens, Mice (C57BL/6JNarl) immunized with OVA protein were sacrificed to acquire splenocytes, followed by a CD8^+^ T cell collection procedure (MojoSort™ Mouse CD8 T Cell Isolation Kit, BioLegend, San Diego, CA, USA). DC2.4 cells were loaded with OVA (100 µg/ml) using an osmotic shock procedure [71] followed by mitomycin C (25 µg/ml) treatment for 30 min and incubation with CD8^+^ T cells for 4 days. The ELISPOT assay was conducted for mouse IFN-γ (Mabtech, Stockholm, Sweden). The reaction was stopped at different time points. Images of the reaction were acquired and analyzed by ImageJ software.

A cytotoxic assay was performed following the manufacturer’s guidelines (Cytotoxicity Assay Kit, carboxyfluorescein succinimidyl ester (CFSE), 7-aminoactinomycin D (7-AAD), Abcam). In brief, target cells (B16F10-OVA) were pre-incubated with CFSE for 15 min then recovered for 30 min. CFSE-labeled B16F10-OVA cells were incubated with OT-1 cells for 1 h followed by staining with 7-AAD for 15 min. Since B16F10-OVA and OT-1 cells were distributed at two distinct regions shown by flow cytometry, only the region that contained B16F10-OVA was selected. The percentage of labeled populations was analyzed by an Attune™ NxT Acoustic Focusing Cytometer (Invitrogen).

### Luciferase assay

Jurkat/NFAT-Luc T cells were seeded 16 h earlier, followed by incubation with different concentrations of human IL-1Ra for an additional 2 h. T cell activation was induced by adding 1.5 µg/ml ionomycin (Sigma-Aldrich) and 1 µM phorbol ester (PMA) (Selleck Chemicals, Houston, TX, USA) for 4 h. Luciferase assay was performed following the manufacturer’s guideline. In brief, the detection reagent, QUANTI-LucTM Gold (InvivoGen) was added to the supernatant of ionomycin/PMA induced-Jurkat/NFAT-Luc T cells. Luciferase activities were measured by a Varioskan Flash spectral scanning multimode reader (Thermo Fisher Scientific, Waltham, MA, USA).

### Bioinformatics and statistical analyses

Expression data of pancreatic cancer and normal pancreas were downloaded from The Cancer Genome Atlas (TCGA) Hub (tcga.xenahubs.net; version: 2019-07-20) and GTExPortal (gtexportal.org/home/datasets; version: 2017-06-05_v8) respectively. Differences between individual groups were analyzed by Student’s *t*-test and presented as the mean ± standard deviation (SD). The overall survival of pancreatic cancer patients was performed by The Human Protein Atlas. Gene programs associated with four subtypes of PDA, including [1] squamous; [2] pancreatic progenitor; [3] immunogenic, and [4] aberrantly differentiated endocrine exocrine (ADEX), and immunogenic populations (e.g., B and T cells) were utilized based on an earlier study [20]. Gene sets were selected into each gene program based on a criterion with a false discovery rate less than 0.1. Correlation coefficients were calculated by Gene Expression Profiling Interactive Analysis 2 (GEPIA2) [72]. p-values below 0.05 were considered statistically significant.

### Electronic supplementary material

Below is the link to the electronic supplementary material.

Supplementary Material 1

## Data Availability

All data generated or analyzed during this study are available from the corresponding author on reasonable request.

## Abbreviations

APCs: Antigen-presenting cells
CTLs: Cytotoxic T lymphocytes
CTLA4: Cytotoxic T-lymphocyte-associated protein 4
ELISPOT: Enzyme-linked immune-absorbent spot
IL-1Ra/IL1RN: Interleukin 1 receptor antagonist
IL1R1: Interleukin 1 receptor 1
IFN-γ: Interferonγ
KRAS: Kirsten rat sarcorma virus
OVA: Ovalbumin
PD-1: Programmed cell death protein 1
PD-L1: Programmed death-ligand 1
PDA: Pancreatic ductal adenocarcinoma
PDX: Patient-derived xenograft
TME: Tumor microenvironment
TP53: Tumor protein 53
