# Supplementary material for: Tumor-derived interleukin-1 receptor antagonist exhibits immunosuppressive functions and promotes pancreatic cancer
Source: Cell Biosci. 2023 Aug 10;13:147. doi: 10.1186/s13578-023-01090-8 (PMC10416534; doi:10.1186/s13578-023-01090-8)
Supplement: Supplementary file 1 — Supplementary Material 1 [file 13578_2023_1090_MOESM1_ESM.docx]

**Additional file 1**

**Title:**

Tumor-derived interleukin-1 receptor antagonist exhibits immunosuppressive functions and promotes pancreatic cancer

**Fig. S1**

**
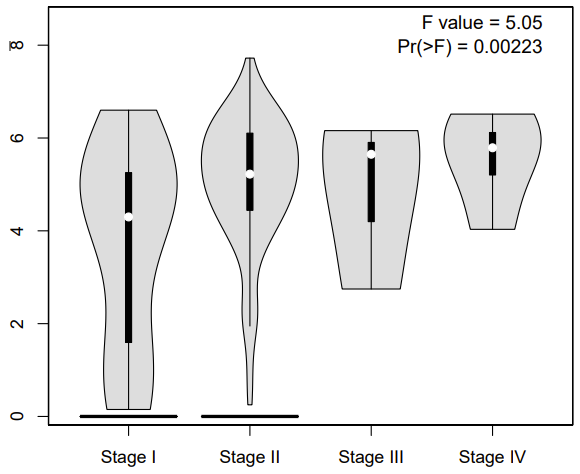
**

**Human *IL1RN* mRNA expression is associated with malignant pancreatic cancer tumor stages.** Stage plot was performed in GEPIA2 website using the human *IL1RN* mRNA level and tumor stages data from TCGA database.

**Fig. S2**

**
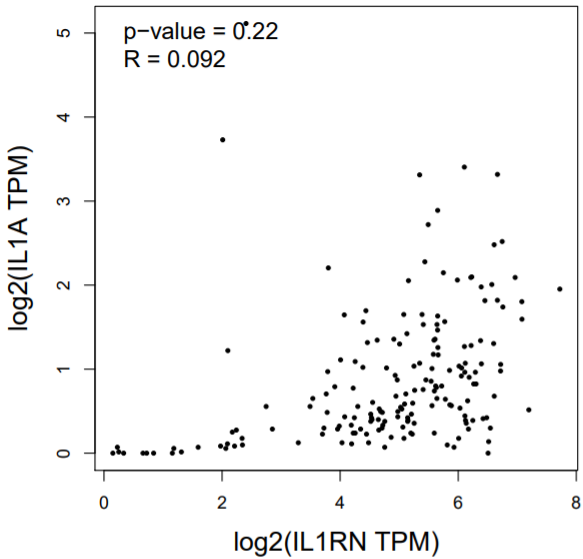

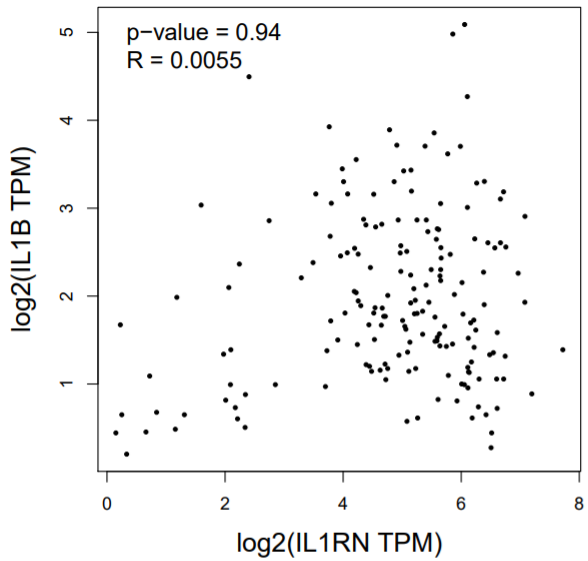
**

**Correlation analysis between *IL1RN* and IL-1α and IL-1β.** mRNA expressions are from the clinical pancreatic cancer dataset deposited in the Cancer Genome Atlas (TCGA). The mRNA levels were analyzed by the GEPIA2 website. R: correlation coefficient using Spearman’s rank correlation. TPM: transcripts per kilobase million. IL1A: IL-1α, IL1B: IL-1β.

**Fig. S3**


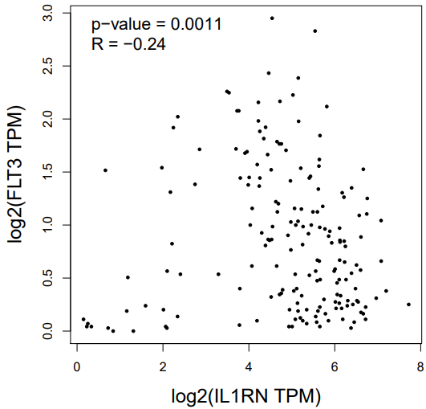

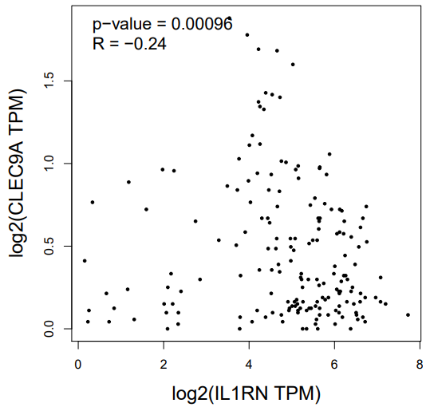

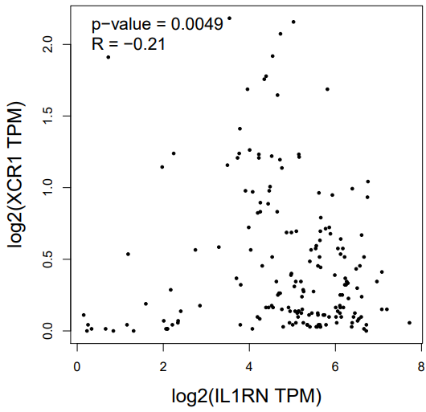
A. B. C.

**Correlation analysis between *IL1RN* and genes involved in cross-presentation.** mRNA expressions are from the clinical pancreatic cancer dataset deposited in the Cancer Genome Atlas (TCGA). The mRNA levels were analyzed by the GEPIA2 website. R: correlation coefficient using Spearman’s rank correlation. TPM: transcripts per kilobase million. XCR1: X-C motif chemokine receptor 1. CLEC9A: C-type lectin domain family 9 member A. FLT3: Fms related receptor tyrosine kinase 3.

**Fig. S4**


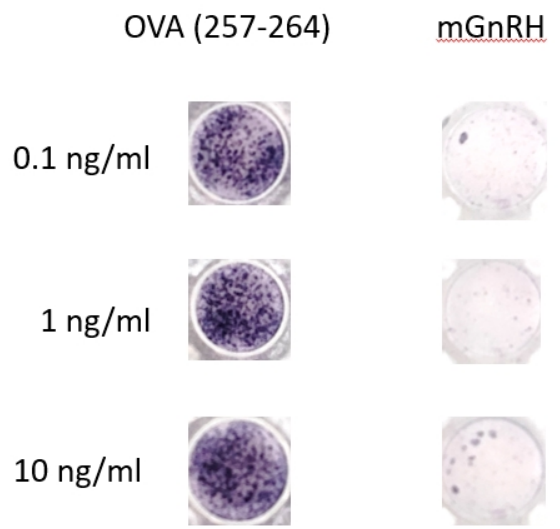


**Monitor the antigen-specific immune response of OVA-specific splenoctyes.** The peptide-induced IFN-γ secretion was compared using ELISPOT. Different dosages of OVA (257-264) and mouse GnRH (mGnRH) peptides were incubated with OT-1 splenocytes.

**Fig. S5**

**
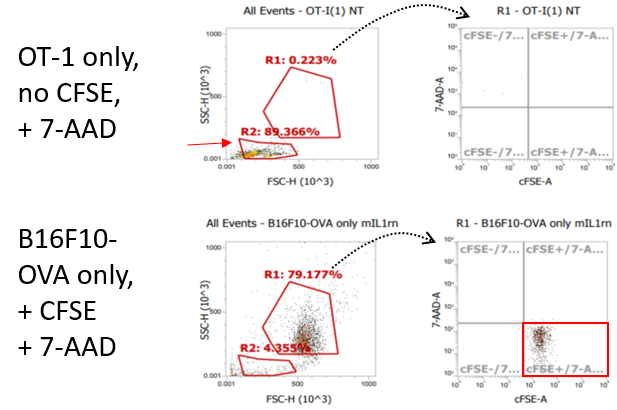
**

**OT-1 cells and B16F10-OVA cells in a mixture can be distinguished by flow cytometry.** OT-1 cells located at R2 region (red arrow, middle panel) but B16F10-OVA cells majorly distribute at R1 region. The R1 region was selected for analyzing CFSE- and 7-AAD-positive cells.

**Table S1.** Antibody information for Western blotting.

| Primary antibody | Application | Source | Dilution | Secondary antibody | Source | Dilution |
| --- | --- | --- | --- | --- | --- | --- |
| Ras (G12D) (D8H7) | Western Blotting | Cell Signaling (#14429S); monoclonal | 1/1000 | anti-rabbit IgG | Jackson Labs | 1/5000 |
| IL-1R1 | Western Blotting | Invitrogen (PA5-47937); polyclonal | 1/1000 | Anti-goat IgG | Jackson Labs | 1/10000 |
| IL-1RA | Western Blotting | Invitrogen (PA5-21776); polyclonal | 1/1000 | anti-rabbit IgG | Jackson Labs | 1/5000 |
| GAPDH | Western Blotting | GeneTex (GTX1000118); polyclonal | 1/1000 | anti-rabbit IgG | Jackson Labs | 1/10000 |
| IL-1RA | IHC | Abcam (ab124962); monoclonal | 1/50 | anti-rabbit | Vector Lab. | 1/1000 |
| Foxp3 | IHC | GeneTex (GTX107737); polyclonal | 1/100 | anti-rabbit | Vector Lab | 1/1000 |

**Table S2.** Primer sequences for the IL1RN-KO.

| **Primer name** | **Primer sequence (5'-3')** | **Function** |
| --- | --- | --- |
| mIL1RN Exon2-F | CAC CGG CCT GCA AGA TGC AAG CCT TC | Cloning |
| mIL1RN Exon2-R | AAA CGA AGG CTT GCA TCT TGC AGG C | Cloning |
| mIL1RN-intron (Exon2)-F | CAG TGA CAT GAC ACT GTC CTT TG | Genomic DNA Sequencing |
| mIL1RN-intron (Exon2)-R | CCT GCT GAA GTA TTC CTC TTT GC | Gemomic DNA Sequencing |
